# Hyperimmunoglobulin syndromes: A review of HIGM, HIES, and HIDS

**DOI:** 10.1016/j.jtauto.2025.100297

**Published:** 2025-06-27

**Authors:** Wushu Chen, Xingpei Li, Junye Mai, Kailang Tang, Yingqiao Wang, Yan-yan Lu, Jie Liang, Ni-jiao Li, Xiu-Yu Qin, Yu Li, Lunkai Yao, Ye Qiu

**Affiliations:** a Department of Gastroenterology and Respiratory Medicine, The Affiliated Tumor Hospital of Guangxi Medical University, Nanning, 530021, Guangxi, China; b Guangzhou Medical University, China

**Keywords:** Hyper-IgM syndrome (HIGM), Hyper-IgE syndrome (HIES), Hyperimmunoglobulin D syndrome (HIDS)

## Abstract

At present, there is a lack of detailed understanding and research on the pathogenesis and treatment of Hyperimmunoglobulin M syndrome (HIGM), Hyperimmunoglobulin E syndrome (HIES), and hyperimmunoglobulin D syndrome (HIDS), and few studies have been conducted to correlate the pathogenesis and treatment of the three disorders. The existing studies are rarely related to the three diseases. We searched PubMed for a large number of relevant literature and analyzed and summarized the contents. We analyzed and introduced the three diseases and their Categorization, Epidemiology, Clinical manifestations, Laboratory diagnosis, and Treatment by means of tables and Figures. It is hoped that this analysis and summary can play a certain role in the research and treatment of related diseases and promote the understanding and prevention of related diseases.

## 1 Introduction

Immunoglobulins (Igs) are globulins with antibody (Ab) activity or a chemical structure similar to that of antibody molecules. They defend against invading pathogens and maintain immune balance and play important roles in the immune system, guarding against invading pathogens and maintaining immunological balance. Immunoglobulins serve a variety of purposes, including antigen recognition and binding, pathogen neutralization, improved phagocytosis, complement system activation, and immune response modulation. Immunoglobulin molecules consist of two identical light chains (L chains) and two identical heavy chains (H chains), which are linked together by disulfide bonds to produce a tetrapeptide chain molecule known as the Ig monomer. Immunoglobulins are categorized into five categories depending on the constant region of the H chain: IgG, IgA, IgM, IgD, and IgE. Each type of immunoglobulin provides distinct protection against pathogens.

IgG: IgG, the most plentiful immunoglobulin in the human body, is mostly contained within blood and tissue fluids. IgG accounts for 75 % of the total immunoglobulins and plays an essential role in fighting against multiple ailments. IgG may eliminate pathogens from the body via a variety of methods, including neutralizing bacterial toxins, inhibiting the bacterial and viral invasion of host cells, and promoting antibody-dependent cytotoxicity. Subsequently, immune cells, such as macrophages and other natural killer cells, are activated to fight pathogens.

IgA: IgA is found largely on mucosal surfaces and in secretions, such as saliva, tears, nasal fluids, breast milk, and intestinal secretions. IgA is structurally similar to IgG, but it has a distinct structural domain known as the "secretory piece" that allows it to interact with mucosal epithelial cells. IgA is the most abundant immunoglobulin in the immune system, comprising approximately 15–20 % of all immunoglobulins in the body. Its major job is to protect mucosal surfaces from harmful bacteria by preventing pathogenic adhesion and invasion, neutralizing microbial toxins, supporting mucosal health, and reducing inflammation.

IgM: The first and most prominent Ig subtype that the body produces, immunoglobulin IgM (IgM), frequently takes on a clumped configuration. Due to its unique structure, large aggregates can develop, which is essential for eliminating a wide range of diseases. IgM consists of five protein subunits, and a J-chain holds its crossing pattern in place. IgM is a monomeric antibody that structurally resembles IgG, with two heavy chains and two light chains at its core. IgM is essential for the early phases of the immune response because it neutralizes viruses, bacteria, and other dangerous microbes. Its multifarious importance in immune protection is highlighted by the fact that, in addition to neutralization, it also activates the complement system, causes inflammation, and increases phagocytosis.

IgE: Mostly involved in the immune response to allergic reactions and parasitic infections, immunoglobulin E (IgE) is structurally similar to other immunoglobulins in both its heavy and light chains. As a result of interactions between IgE and eosinophils and mast cells, allergic symptoms arise. An allergic reaction is caused by the body's reaction to an allergen, which is caused by a particular IgE binding to the allergen and activating mast cells and eosinophils, which release histamine and other mediators. Allergy-related illnesses, such as hay fever and asthma, have been linked to elevated IgE levels.

IgD: The specific structural and functional details of immunoglobulin D (IgD), a very uncommon subclass of immunoglobulins, are unknown, but IgD is present on the surface of B lymphocytes. Recent investigations have started to shed light on the function of IgD in B-cell activation and modulation as well as its interactions with other immune components to control the production of antibodies. IgD may also contribute to hypersensitivity reactions, including the production of antibodies against penicillin, insulin, nuclear antigens, "O" antigens, basement membranes, and autoantibodies linked to autoimmune diseases, such as systemic lupus erythematosus, thyroiditis, and rheumatoid arthritis.

In the delicate balance of immune harmony, complex interactions between normal concentrations of various immunoglobulins protect physiological integrity. However, this shift in balance can cause gaps in our immune system structure, resulting in primary immunodeficiency disease (PID), a genetic illness defined by a weakness in the immune system's ability to protect itself against invading microorganisms. As a result, it is vital that we do not dismiss these disorders due to their low incidence rates but rather expand our awareness and research to find strategies to prevent future invasions and defend vulnerable individuals. Through thorough literature review and statistical analysis, we have gained a better understanding of the three most commonly occurring disorders, hyperimmunoglobulin M syndrome (HIGM), hyperimmunoglobulin E syndrome (HIES), and hyperimmunoglobulin D syndrome (HIDS), and have written this review. This comprehensive review aims to provide a deeper understanding of these disorders and draw attention to their importance. By shedding light on these conditions, we hope to stimulate further research and promote a more focused approach toward addressing these disorders.

**Fig. 1:**
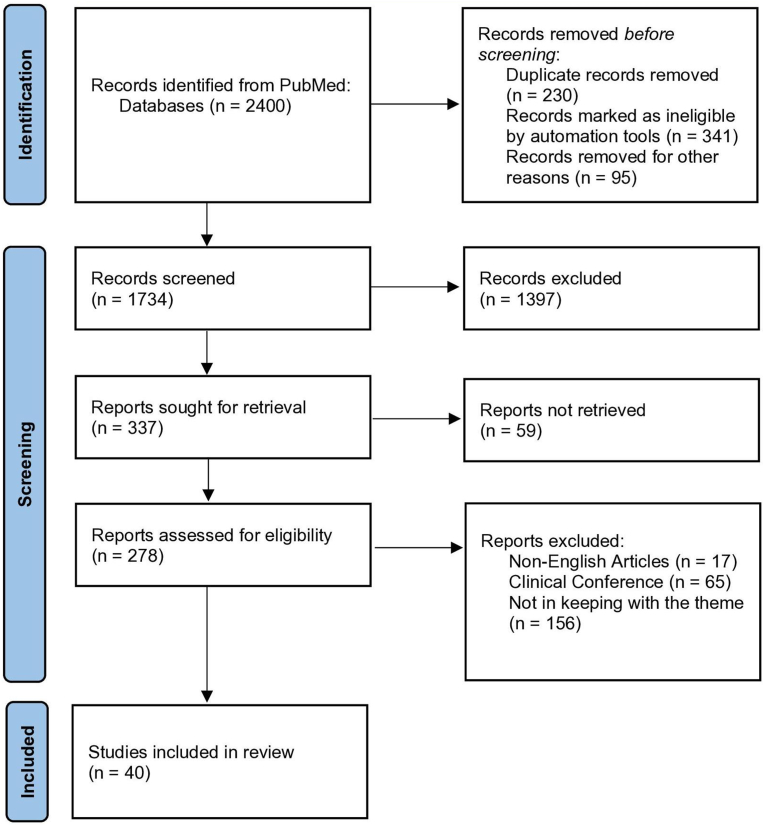
PRISMA Flow Diagram of Literature Screening Process. Records identified from PubMed (n = 2400). After duplicate removal (n = 666), 1734 records underwent initial screening by two independent authors using titles/abstracts. Discrepancies resolved by third author consensus. Full texts of potentially eligible studies were assessed by two additional authors applying inclusion/exclusion criteria. Final included studies: n = 40.

To provide this deeper understanding and draw attention to the importance of these disorders, we conducted a systematic review of the literature following established guidelines. The methodology employed for identifying and selecting relevant studies is described here:

A total of 2400 studies were obtained through the PubMed database, and 1734 potential studies were identified in the first phase. All articles were imported into a file manager (Endnote 20, Clarivate Analytics, Philadelphia, USA), then two authors (XL and WS) screened the files by reviewing the title and abstract after dropping the duplicates. Any disagreement would be handed to a third author (KT) and reached consensus after discussion. In subsequent, authors (YQ and JM) would conduct the second screening based on the full text, and the inclusion and exclusion criteria were applied. The Preferred Reporting Items for Systematic Reviews and Meta-Analysis (PRISMA) flow diagram of the search results and article exclusion details are shown in Fig. 1.

## 2 HIGM

### 2.1 Introduction

Hyperimmunoglobulin M syndrome (HIGM) encompasses a group of disorders characterized by normal to elevated levels of serum IgM and significantly reduced or undetectable levels of IgG, IgA, and IgE [1]. The association between HIGM and immunodeficiency was initially established by Charles Janeway, a prominent American immunologist, in 1953. In 1961, Rosen et al. provided a comprehensive description of HIGM [2]. Notably, Danish immunologist Michael B. Jacobsen's seminal research paper published in 1982 contributed significantly to our understanding of the clinical manifestations and genetic basis of HIGM. Jacobsen's work has played a pivotal role in advancing diagnostic and therapeutic approaches for this condition. Molecular elucidation of HIGM occurred in 1992, when a mutation in the CD40 ligand (CD40L) gene was identified [3]. HIGM is classified as a rare inherited primary immunodeficiency disease (PID) known as immunoglobulin class switch recombination (Ig-CSR) deficiency [4,5]. All variants of HIGM syndrome share a common feature of significantly diminished levels of switched immunoglobulins in the bloodstream, coupled with normal or elevated levels of IgM. Individuals affected by HIGM exhibit a decreased population of memory B cells that have undergone isotype switching, while their overall B-cell count remains within the normal range [6]. In patients with HIGM, the typical transformation process by which B cells create different immunoglobulins (e.g., IgM, IgG, and IgA) after infection is disrupted. This situation is caused by genetic abnormalities in the immune system, which result in high IgM levels and decreased levels of other immunoglobulins in the body. As a result, the immune system's ability to properly combat bacteria, viruses, and other disease-causing invaders is impaired. As a result, people with HIGM are at risk for recurring bacterial and fungal infections, which largely impact the respiratory and gastrointestinal systems.

**Fig. 2:**
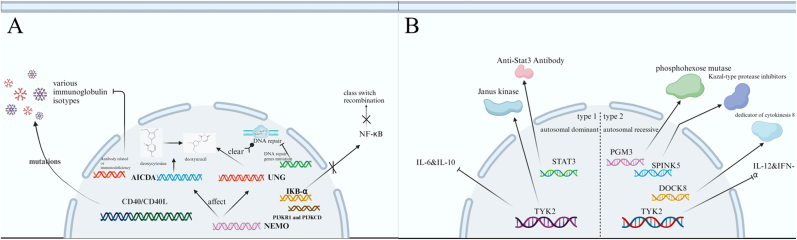
HIGM, HIES-related pathogenic genes. (A) Molecular Mechanisms Underlying Hyper-IgM Syndrome: CD40/CD40L Interaction Defects, B-Cell Intrinsic Deficiencies (AID/UNG), and Dysregulated Signaling Pathways (PI3K/NF-κB) (B) Signaling Pathway Defects in Hyper-IgE Syndrome: STAT3, TYK2, and DOCK8 Dysregulation Drive Cytokine Impaired Signaling in AD-HIES and AR-HIES. Abbreviations: UNG= Uracil-N-Glycosylase; NEMO = NF-Kappa-B essential modulator; IKB-α = NF-kappa-B inhibitor alpha; AICDA = activation-induced cytidine deaminase gene; CD40 = CD40 molecule gene; CD40L = CD40 ligand; NF-κB = Nuclear factor-k-gene binding; PGM3 = Phosphoglucomutase 3; SPINK5 = Serine Peptidase Inhibitor Kazal Type 5; DOCK8 = Dedicator of Cytokinesis 8; TYK2 = Tyrosine Kinase 2; IL-6 = Interleukin 6; IL-10 = Interleukin 10; IL-12 = Interleukin 12; IFN-α = Interferon-α

The underlying cause of HIGM involves the failure of B cells to undergo maturation through immunoglobulin isoform switch recombination (CSR) and somatic hypermutation (SHM). This impairment can be attributed to defects in the CD40L‒CD40 pathway or deficiencies in the enzymes essential for CSR and SHM, which are crucial for B-cell activation [7]. The CD40L‒CD40 interaction plays a pivotal role in B-cell class switch recombination (CSR) and somatic hypermutation (SHM) by orchestrating T/B cell collaboration through two interconnected pathways: (1) direct T-B contact, which delivers cytokine-guided CSR signals and promotes plasma cell differentiation, and (2) DC-mediated priming that enhances antigen presentation, amplifies Th cell activation, and coordinates lymphoid recruitment of antigen-specific lymphocytes, ultimately driving the generation of diverse immunoglobulin isotypes against pathogens [8,9]. In addition to mutations in the CD40 and CD40L genes, HIGM syndrome may be caused by mutations in the AICDA and UNG genes [6,[10], [11], [12], [13], [14], [15]], the PI3KR1 and PI3KCD genes [10,[16], [17], [18], [19], [20], [21], [22], [23], [24], [25]], the NEMO and IKB-α genes [7,10,[26], [27], [28], [29], [30], [31], [32], [33], [34]], DNA repair gene mutations [10,25,[35], [36], [37], [38], [39], [40], [41], [42], [43], [44], [45]], and mutations in other genes [37,[46], [47], [48], [49]] (Fig. 2(A)).

### 2.2 Categorization

Due to uncertainty regarding the etiology of HIGM and the site of mutation, HIGM can be broadly categorized into seven subtypes, namely, X-linked HIGM [2,4,7,10,[50], [51], [52], [53], [54], [55]], HIGM2, HIGM3 [7,50,56], HIGM4 [57], HIGM5 [7] HIGM6, and HIGM7 [7,28] (Table 1). Approximately 25 % of HIGM patients have normal CD40L, CD40, AID, and UNG genes, and some of these patients have been identified as having defects in CSR [7,58].

**Table 1:** Classification of HIGM subgroups by genetic mechanisms and pathophysiological characteristics.

Subtype	Features, characteristics
X-linked HIGM	X-linked HIGM is the most common subtype (65–70 %) and affects the immune function of T and B cells due to defective T-cell costimulation caused by mutations in the CD40L gene. In addition, more than 100 different mutations, such as single amino acid substitutions, truncations, and in-frame or out-of-frame deletions, are present in patients with x-linked HIGM.
HIGM2	HIGM2 is an autosomal recessive variant caused by mutations in the activation-induced cytidine deaminase gene (AICDA), which manifests as enlarged tonsils, enlarged lymph nodes, and recurrent bacterial sinus infections.
HIGM3	HIGM3 is an autosomal recessive variant caused by mutations in the CD40 gene (TNFRSF5) and exhibits clinical features indistinguishable from those of patients with defects in the CD40L gene.
HIGM4	The genetic cause of HIGM4 is unknown. Patients with HIGM4 represent the least characterized subgroup of patients whose clinical presentation resembles the milder form of HIGM2 and produces residual IgG.
HIGM5	HIGM5 patients exhibit clinical similarities to HIGM2 patients, but they carry mutations in the uracil DNA glycosylase gene (UNG). Mutations in AICDA and UNG are believed to be intrinsic B-cell defects as they encode enzymes involved in CSR and SHM
HIGM6	HIGM6 is caused by heterozygous mutations in the NF-κB essential regulator (NEMO/IKKG) gene and is mainly characterized by the development of hypogammaglobulinemia and hypohidrotic ectodermal dysplasia in male patients.
HIGM7	HIGM7 is related to IκBα, a related molecule of the HIGM6 mutation pathway, resulting in the translocation of NF-κB to the nucleus, leading to aberrant expression of several enzymes, including AID and UNG.

∗ X-linked HIGM can be traced back to two brothers with a history of recurrent bacterial infections in 1961 [2].

### 2.3 Epidemiology

**Fig. 3:**
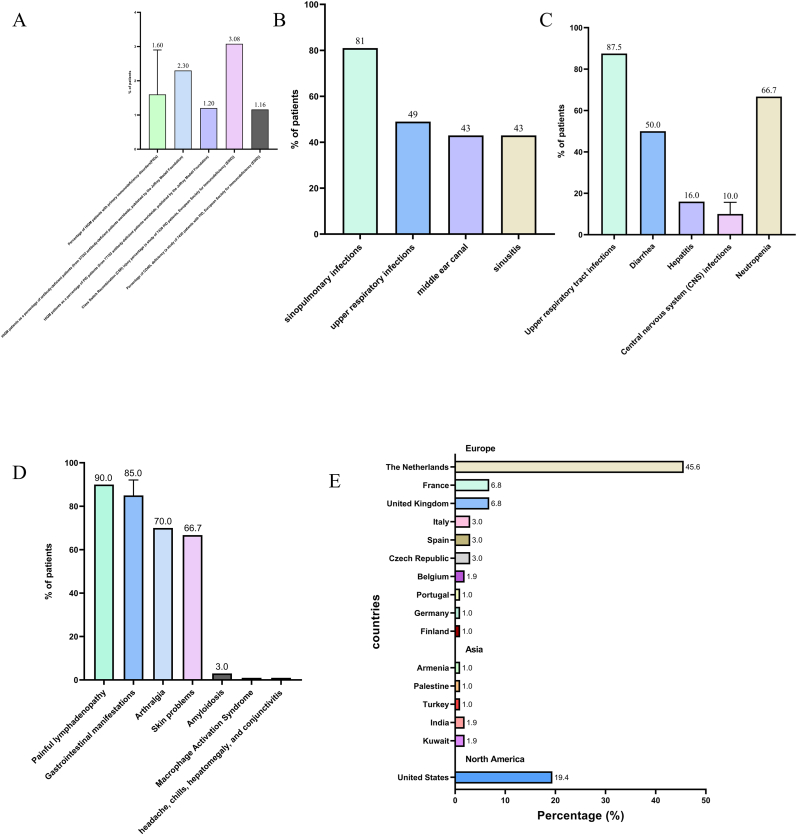
Epidemiology of HIGM, HIDS or complications of infection. (A) Prevalence of HIGM across studies. (B) A North American cohort study of 79 patients with X-HIGM. (C) Clinical manifestations of HIGM. (D) Clinical manifestations of HIDS. (Macrophage activation syndrome and headache, chills, hepatomegaly, and conjunctivitis are shown as isolated occurrences only.) (E) Sources of the 103 HIDS patients included in the study.

Because HIGM is a rare congenital immunodeficiency disorder, we know very little about HIGM. An in-depth study of the epidemiologic features of HIGM is an important way to understand HIGM, which usually manifests in infancy or early childhood (depending on variables such as mutation type, disease severity, and individual immune response); therefore, the vigilant monitoring of symptoms, such as altered immune function and recurrent infections, in infants and children with a family history of HIGM is necessary for timely diagnosis and intervention. The following is an example of a family history of HIGM. Although the incidence of HIGM is low [59], the prevalence of HIGM varies in different parts of the world [[59], [60], [61], [62], [63]], data indicate that the United States reported most of the cases (for example, in the subtype of CD40L deficiency, there are 272 cases in the US, accounting for approximately 73 % of the global total) [59], whereas Iran reported only 28 cases [60]. In addition to differences in the prevalence of HIGM in different regions, differences in the prevalence of HIGM among different ethnic and racial groups have also been documented [25,64]. The prevalence of HIGM varies greatly by sex, with the subtype with the highest percentage of X-linked HIGM being much more prevalent in men than in women [55,63]. Although HIGM is uncommon, it can be fatal [65](Fig. 3(A and B)).

### 2.4 Clinical manifestations

Patients with HIGM exhibit a unique immunologic profile of elevated levels of IgM and reduced levels of IgG and IgA, resulting in an impaired immune response to infection. Although the disease is rare in incidence and prevalence, its morbidity and mortality are high. The most common form of HIGM is X-linked HIGM, which usually stems from a defect in CD40L/CD40. This defect makes patients highly susceptible to a variety of pathogens, including bacteria, fungi, viruses, and parasites, such as Pneumocystis (carinii) jiroveci, Bartonella, Cryptosporidium parvum (resulting from exposure to contaminated drinking water), and Histoplasma [[66], [67], [68], [69], [70], [71], [72], [73], [74]]. Respiratory tract infections, especially pneumonia, were the major organ-specific complication in patients with HIGM in North American and European studies [63,65]. Gastrointestinal infections are another major burden, which often manifest as diarrhea and lead to malnutrition and other complications. The most common pathogen to cause these symptoms, Cryptosporidium, also leads to sclerosing cholangitis. Hepatitis and cirrhosis may also occur [63,65,75]. Although central nervous system infection is uncommon, it carries a serious risk and may lead to long-term neurologic complications [63,[76], [77], [78], [79]]. Hematologic abnormalities, such as anemia and neutropenia, are common in HIGM, and the increased risk of malignancies, such as lymphoma, complicates the clinical picture. These multifaceted challenges highlight the fact that patients with HIGM are vulnerable to a variety of infections because of dysregulated immunoglobulin secretion [63,65,75,[80], [81], [82]] (Table 2) (Fig. 3(C)).

**Table 2:** Organ system-based presentation of infectious manifestations and etiology in Hyper-IgM syndrome (HIGM).

Infectious System	Infections/Complications (%)	Pathogen/Causes
Lung infection (North American Report of X-HIGM)	Pneumonia (81 %)	
	Upper respiratory infections (49 %)	
	Middle ear canal (43 %)	
	Sinusitis (43 %)	
Lung infection (European Report of X-HIGM)	Upper respiratory infections (87.5 %)	
	Subsequent development of bronchiectasis (82.5 %)	
	Parenchymal (51.8 %)	
	Interstitial infections (39.3 %)	
Intestinal infection	Diarrhea (50 %)	Cryptosporidium
		*Giardia lamblia*
		Rotavirus
		*C. difficile*
		Yersinia
	Sclerosing cholangitis	Cryptosporidium
	Hepatitis (16 %)	Hepatitis B
		Hepatitis C
		CMV
	Liver cirrhosis	Fresh frozen plasma/Blood transfusions
Central Nervous System Infections	Central Nervous System Infections (6–14 %), such as cryptococcal meningoencephalitis (CM)	*Toxoplasma gondii*
		Echovirus
		*Cryptococcus neoformans*
		Cytomegalovirus
		*Mycobacterium bovis*
		*Streptococcus pneumoniae*
		John Cuningham (JC) Virus
		Pneumococcus
		ECHO virus
		Cryptococcus
Hematologic manifestations	Neutropenia (2/3 in XHIM)	Gingivitis
		Anal ulcers
	Anemia (1/3 in XHIM)	Chronic infections
		Parvovirus B19
	Significant enlargement of tonsils, lymph nodes, spleen, and liver	Lymphoid hyperplasia
Malignancies	Lymphomas	
	Liver, pancreas, biliary tree, and neuroectodermal endocrine cell carcinoma	

### 2.5 Laboratory diagnosis

The laboratory indicators of HIGM are neutropenia, radiosensitivity, serum alpha fetoprotein levels [10], switching memory B cells [56], tandem antigen-induced T cells that exhibit defects in proliferation [3,65,[83], [84], [85], [86]], activated PI3K signal transduction products [[18], [19], [20], [21], [22], [23], [24], [25],^86^], CD40L expression [4,[87], [88], [89], [90], [91]], and the soluble ecd40-ig fusion protein [48], as indicated by cell fluorescence studies [92] and genetic evaluation [[93], [94], [95]] (Table 3).

**Table 3:** Laboratory diagnosis of Hyper-IgM syndrome: Characteristic patterns of key indicators.

Targets	Features, characteristics
Neutropenia	Observed neutropenia is common in patients with X-HIGM
Radiosensitivity	Increased radiosensitivity observed in patients with DNA repair defects
Serum Alpha fetoprotein Levels	Elevated serum alpha-fetoprotein levels in patients with ATM defects
Switching Memory B Cells	Significantly reduced number of switching memory B cells in CD40 and CD40L-deficient patients
Tandem antigen-induced T cells exhibit defects in proliferation	Some XHIGM gene deficient patients exhibit deficits in tandem antigen-induced T-cell proliferation
Activated PI3K Signal Transduction Products	Activated PI3K signaling products such as phosphorylated AKT and phosphorylated S6 in APDS patients serve as potential diagnostic markers
CD40L Expression	Detection of CD40L expression deficiency was assessed by stimulation of T cells followed using methods such as flow cytometry
Genetic Evaluation	Genetic evaluation should be based on the observation of CD40L gene abnormalities by techniques such as genome sequencing and PCR amplification
The soluble ecd40-ig fusion protein	Most X-HIGM patients can be recognized by looking for soluble ecd40-ig fusion proteins
Cell fluorescence studies	Fluorescence studies of cells from HIGM patients show lack of cd40 expression on the surface of B cells

∗ Normal number of CD27^+^ memory B cells in patients with AID and UNG deficiency.

∗ Peripheral blood T cells (including CD4^+^ and CD8^+^ T-cell subsets) as well as T-cell proliferation in AID and UNG-deficient patients were within the normal limits.

∗ Patients may not be identified by flow cytometry screening techniques even in the presence of in-frame deletions or insertions.

∗Cytofluorescence studies should consider the possibility of false-positive results, as anti-CD40 monoclonal antibodies can also recognize functionally defective CD40 due to heterozygous mutations in the CD40 gene or mutations in CD40 expressed on the cell surface.

### 2.6 Treatment

After testing and diagnosing patients with HIGM, appropriate treatments, such as supportive therapy, total parenteral nutrition [96], antibiotic strategies, antifungal drugs, immunoglobulin replacement therapy (IVIG) [65,[96], [97], [98]], recombinant G-CSF therapy, bone marrow transplantation [75,99], stem cell transplantation, and gene therapy [10], are provided as appropriate (Table 4).

**Table 4:** Hyper-IgM syndrome (HIGM): Treatment approaches, clinical significance, and specific therapies.

Treatment method	Purpose	Specific treatments
Supportive therapy	Ensure an optimal blood volume status	Primarily through oral rehydration and selective use of antidiarrheal agents.
Total parenteral nutrition	Provide active nutritional support	
Antibiotic strategy	Against pathogens such as Pneumocystis carinii and Bartonella spp.	Provide appropriate antibiotics primarily for pathogens in the patient's body
Antifungal drugs	Treatment-related fungal complications	Provide antifungal drugs such as fluconazole, itraconazole, amphotericin B according to the patient's specific situation
Immunoglobulin replacement therapy (IVIG)	Provides deficient antibodies needed to prevent and control infections/reduces elevated IgM levels to ameliorate neutropenia	The recommended dose is 400–600 mg/kg IVIG every 21 days
Recombinant G-CSF therapy		In cases where neutropenia persists despite IVIG treatment, recombinant G-CSF therapy may be beneficial
Bone marrow transplantation	Restoration of normal immune function	When the patient's level of morbidity is severe, the patient's abnormal hematopoietic stem cells are replaced with healthy hematopoietic stem cells to restore normal immune function.
Stem cell transplantation	Restoration of normal immune function	Replacement of abnormal hematopoietic stem cells by infusion of healthy hematopoietic stem cells, thus restoring normal immune function
Gene therapy	Replacement of disease-causing genes	Providing targeted gene therapy based on identified causative genes, particularly in the context of activated phosphoinositide 3-kinase delta syndrome (APDS) and DNA repair defects

∗ Antibiotics must be used with caution because patients with HIGM have an abnormal immune system. Doctors tailor a personalized antibiotic regimen based on the patient's specific situation, including immune status, medical history and potential drug resistance.

∗ In summary, for different HIGM patients, appropriate treatments should be selected according to their specific conditions and medical recommendations. Continued research and advances in stem cell transplantation and gene therapy are expected to improve the prognosis and quality of life of HIGM patients.

## 3 HIES

### 3.1 Introduction

More than 10 years after HIGM was first identified, Davis and Wedgwood published an initial account of a condition affecting two girls that was marked by recurrent staphylococcal abscesses, pneumonia, and an eczematoid rash appearing in the neonatal period [100,101]. Notably, at the time of their report, the significance of IgE levels had not been recognized [102]. Subsequent investigations by Buckley et al. expanded upon this syndrome [103] by highlighting the associations among recurrent staphylococcal abscesses, chronic eczema, and markedly elevated serum IgE concentrations. Importantly, they reported that the levels of other immunoglobulins remained within normal ranges. Over time, the multisystemic nature of hyperimmunoglobulin E syndrome (HIES) has become evident because manifestations extend beyond immune system abnormalities to include skeletal and connective tissue irregularities, such as scoliosis, osteoporosis, and hyperextensive joints, along with dental anomalies [104]. Subsequent studies in 2004 revealed a novel autosomal recessive form of HIES in consanguineous families [105], whereas in 2006, a deficiency in tyrosine kinase 2 (TYK2) was linked to susceptibility to intracellular bacterial and viral infections in AR-HIES patients [106]. Finally, in 2007, dominant-negative mutations in the signal transducer and activator of transcription 3 (STAT3) gene emerged as major molecular determinants of classical HIES [107,108]. These findings represent the evolution of the understanding of HIES. HIES, known colloquially as Job's syndrome or Buckley's syndrome, was initially identified as a rare primary immunodeficiency disorder. It manifests with recurring staphylococcal skin and pulmonary abscesses, eczematoid dermatitis, notably elevated serum IgE levels, eosinophilia, diminished neutrophil chemotaxis, and variable impairment in T-cell activity [100,103]. In a comprehensive analysis, HIES is intricately categorized into two distinct types, regardless of its inheritance pattern [100,103,[105], [106], [107], [108], [109], [110], [111], [112], [113], [114], [115], [116], [117], [118], [119], [120], [121]] (Table 5) (Fig. 2(B)).

**Table 5:** Hyper-IgE syndromes (HIES): Subtypes, causative genes, and clinical features.

Subtype	Related gene	Features, characteristics
Type 1 HIES (AD-HIES, basic)	STAT3 mutations are found in the majority of patients with type 1 HIES and defects in the IL-6 and IL-10 signaling pathways	Type 1 HIES presents with systemic abnormalities, including the skeletal and dental systems, often leading to pulmonary cyst formation and predisposing to concurrent Aspergillus and multidrug-resistant *Pseudomonas aeruginosa* infections.
Type 2 HIES (AR-HIES)	TYK2 deficiency is the molecular etiology of Type 2 HIES and defective IL-12 and IFN-α signaling	Patients with Type 2 HIES do not have skeletal abnormalities, but recurrent viral infections, such as molluscum contagiosum and herpes simplex virus (HSV) infections, are common. The presence of gas puffing is not usually observed in such patients, and most patients exhibit minor defects in signaling downstream of the T-cell receptor complex.

### 3.2 Epidemiology

HIES, like HIGM, is a rare immunodeficiency disease with a very low incidence. The occurrence of HIES does not differ by ethnicity or sex, and HIES mostly affects newborns and young children, with an incidence of fewer than 1 per 100,000 [122]. Across independent cohorts, STAT3 mutation prevalence in HIES patients differed substantially: one study identified mutations in 64 of 100 patients (64 %), whereas another observed a ratio of 4 in 14 (29 %). [123,124]. The estimated yearly incidence of AD-HIES is estimated to be one in one million [125]. Due to this very low incidence, which we know little about this condition. In India, the first patient with HIES was recorded only in 1994, and only a few cases have been reported in the following years. According to a study conducted by Gupta et al., in 2012 (reference 26), the prevalence of HIES was found to be 4.9 % and 16.3 % among all diagnosed cases of immune system disorders (IEIs) at two prominent medical centers in India [126]. In July 2016, the USIDNET Registry boasted an extensive dataset containing information on 85 patients diagnosed with HIES. Among the patients, 50 were male, whereas 35 were female, representing a diverse cohort. Remarkably, these individuals hailed from 25 distinct states across the United States, with additional representation from the province of Quebec. The registry records spanned an impressive 15-year period, commencing in 2001 and ending in 2016, providing valuable insights into the long-term progression of this condition [125].

### 3.3 Clinical manifestations

HIES patients have normal serum IgM, IgG, and IgA levels, but many patients have various deficiencies in the antigen-specific antibody response to immunity [127]. In addition, they are susceptible to infection by enveloping bacteria [[128], [129], [130], [131]]. Deficiencies in IgG subclasses have been documented in some patients [127]. In addition, patients with mutations in the HIES and STAT3 genes tend to have lower than normal numbers of circulating memory B cells [132]. An extensive analysis of 103 patients with HIES revealed that, like HIGM patients, HIES patients have an abnormal immune system due to dysregulated levels of immunoglobulins in their bodies, which makes them susceptible to a variety of infections caused by a variety of pathogens, including bacteria and fungi [133]. As shown in a study associated with 82 HIES patients, unlike HIGM, HIES infections primarily affect the lungs and skin [125] (Table 6).

**Table 6:** Infectious complications in Hyper-IgE syndrome (HIES): Research group profiles with symptom and pathogen distribution.

Research Groups	Infections/Complications (%)	Detailed symptoms/pathogens
A study involving 103 patients with HIES	Concomitant candidiasis (approximately 50 %)	Includes oral candidiasis (most prevalent), nail candidiasis, pulmonary candidiasis, and nail fungal diseases
	Porter's spondylitis	Developed subcutaneous abscesses on their arms, with acid-resistant bacilli detected in the pus aspirates
	Anal and oral ulceration	
	Cytomegalovirus pneumonia	
A study involving 82 patients with HIES	Localized skin abscesses (74.4 %)	
	Pneumonias (72 %)	Mainly caused by *Staphylococcus aureus* (72.3 %) or *Aspergillus fumigatus* (27.4 %)
	Infected pneumatoceles (19.5 %)	Mainly caused by *Aspergillus fumigatus* (57.1 %) and *Pseudomonas aeruginosa* (35.7 %)
	Skin abscesses (74.4 %)	
	Herpes infection (7.4 %)	
	A case of Molluscum contagiosum	
	A case of fever associated with an Epstein‒Barr virus infection	
In an allergy-related study involving 82 patients with AD-HIES	Food allergies (37 %)	
	Environmental allergies (18.3 %)	
	Drug allergy (42.7 %)	

Patients diagnosed with HIES typically present with early pustules and eczema, which are often associated with *S. aureus* colonization in infancy. These dermatologic manifestations often persist into childhood and are most effectively treated with topical or systemic antistaphylococcal therapy [[134], [135], [136], [137], [138], [139]]. In HIES, STAT3 mutations disrupt STAT3 phosphorylation and impair Th17 cell generation. Th17 cells are critical for producing IL-17A, IL-17F, and IL-22, cytokines essential for inducing antimicrobial peptides (AMPs) in epithelial cells at mucosal and skin surfaces [8,9], rendering them more vulnerable to cutaneous Candida infections of the mucous membranes, with approximately 70 % of patients experiencing chronic mucocutaneous infections [125,138,140]. Furthermore, lung infections in individuals with HIES often present as recurrent septic pneumonia, lung expansion issues, bronchiectasis, and fungal infections [101,104,110,127,134,138,[141], [142], [143], [144], [145], [146], [147]]. Notably, HIES patients are susceptible to coinfections [125]. To determine the potential reasons for the susceptibility the lungs and skin of HIES patients to infection, researchers have examined the expression of chemokines and antimicrobial peptides in different types of immune and nonimmune cells. An investigation revealed that keratinocytes (skin) and bronchial epithelial cells (lungs) require a combination of TH17 cytokines and classical proinflammatory cytokines, which work synergistically to produce antistaphylococcal factors. Conversely, fibroblasts, endothelial cells, and macrophages, among other cell types, need only common proinflammatory cytokines to generate these factors [148,149].

In addition to increased susceptibility to invasive infections by pathogens, individuals with HIES are also predisposed to other organ or tissue abnormalities, including craniofacial malformations [104,127,[150], [151], [152]], musculoskeletal anomalies [104,134,139,153,154], dental irregularities [104,155,156], allergies [125,139] and malignant tumors [138,[157], [158], [159], [160]] (Table 7).

**Table 7:** Hyper-IgE syndrome (HIES): Systemic spectrum of infections, pathogens, and symptoms.

Infectious System	Infections/Complications (%)	Pathogen/Causes	Symptom
Skin	Rash	*Staphylococcus aureus*	Develop a pustular and eczematous rash on their face and scalp
	Mucocutaneous Candida infection		Disruptions to the oral microbiota and proliferation of Candida
	Chronic mucocutaneous infections (70 %)		Oral or genital thrush, or onychomycosis
Lung	Recurrent septic pneumonia	*Staphylococcus aureus*, *Streptococcus pneumoniae*, and *Haemophilus influenzae*	
	Purulent pneumonia		Lung expansion issues and bronchiectasis occurring
	Fungal lung infections	Aspergillus	Manifesting as chronic pulmonary aspergillosis (CPA) and allergic bronchopulmonary aspergillosis (ABPA).
Face			Presenting with asymmetry, a broad nose, deep-set eyes accompanied by a prominent forehead and Rough, porous texture
Musculoskeletal abnormality	Minor traumatic fractures	Abnormal osteoclast-mediated bone resorption	Often involving the ribs and long bones
	Degenerative joint disease	Repetitive hyperextension of both major and minor joints	
	Scoliosis		
	Reduced bone density		
Toothlessness	Natural shedding of their primary teeth		
	Distinct alterations in the oral mucosa, tongue, palate, and cheeks	Candida	Such as a central depression of the tongue and a central band of the palate
Allergy	Allergy		Includes allergies to food, drugs, and the environment
Malignant tumor	Lymphomas		Including non-Hodgkin's lymphomas (NHL), T-lymphocyte lymphoma, Hodgkin's lymphoma, B-lymphocyte
	Hodgkin's lymphoma		
	Leukemia		
	Vulvar cancer		
	Hepatocellular carcinoma		
	Lung cancer		

### 3.4 Laboratory findings

Elevated serum IgE and eosinophilia represent the most prevalent laboratory irregularities in HIES [104,127,134]. Furthermore, a distinctive feature involves diminished numbers of memory T lymphocytes and B lymphocytes [161,162] (Table 8).

**Table 8:** Diagnostic laboratory characteristics of HIES: Key biomarkers and clinical significance.

Targets	Features, characteristics
Elevated serum IgE	Age-adjusted elevated IgE levels may be evident in umbilical cord blood at birth, often exceeding 2000 IU/mL.
Eosinophilia	Eosinophilia is frequent but not correlated with IgE levels.
Diminished numbers of memory T-lymphocytes and B-lymphocytes	a distinctive feature involves diminished numbers of memory T-lymphocytes and B-lymphocytes; the development of B-lymphocytes into memory B-lymphocytes relies on pathways transduced through STAT3, including IL-21 and follicular T-lymphocyte subpopulations

∗ Serum IgG, IgA, and IgM typically remain within normal ranges, although mild deficiencies may be observed in some individuals.

### 3.5 Treatment

Treatment for HIES, a rare primary immunodeficiency disorder, focuses primarily on controlling symptoms and preventing complications. Specific treatments include supportive and preventive skin care and antibiotics [139,[163], [164], [165]], hematopoietic stem cell transplantation (HSCT) [139,[166], [167], [168]] and systemic INF-a 2b therapy [169,170] (Table 9).

**Table 9:** Management of Hyper-IgE syndrome (HIES): Therapeutic categories and corresponding interventions.

Treatment method	Specific treatments
Antibiotic therapy	Antibiotics are usually used to treat and prevent bacterial infections, and prophylactic antibiotics may be used for long periods of time to reduce the risk of recurrent infections, e.g., methotrexate/sulfamethoxazole is effective in reducing the incidence of septic pneumonia
Skin care	The use of disinfectants, such as bathing in 120 cc of bleach for 15 min three times a week or swimming in chlorinated pools, is the most effective treatment for eczema and prevention of *Staphylococcus aureus* abscesses
Hematopoietic stem cell transplantation (HSCT)	Hematopoietic stem cell transplants may be an important treatment option for those with serious conditions such as DOCK8 deficiency. Improved immune and nonimmune features of the underlying disease can occur after transplantation.
Systemic INF-a 2b therapy	Systemic INF-a 2b therapy, which hinders viral replication and activates effector lymphocytes, has exhibited efficacy in treating severe viral infections

∗ Because cutaneous staphylococcal infections can worsen the severity and scope of atopic dermatitis, patients with AD-HIES are advised to use systemic antistaphylococcal medicines in addition to conventional topical treatments [164].

∗ Since certain illness symptoms arise from nonhematopoietic sources, the function of hematopoietic stem cell transplantation (HSCT) in LOF STAT3 is complicated [138].

∗ While awaiting definitive transplant therapy, it is critical to manage serious problems associated with infection and cancer, prophylactic use of antimicrobial agents and antiviral medications is recommended, and the use of immunoglobulin replacement therapy should be seriously considered.

## 4 HIDS

### 4.1 Introduction

Hyperimmunoglobulin D syndrome (HIDS) is an exceptionally rare autosomal recessive autoinflammatory disorder, distinguished by recurrent febrile episodes and systemic inflammation. Initially described in 1984 as "hyperimmunoglobulin D and periodic fever syndrome" in a Dutch cohort, the condition was primarily associated with persistently elevated serum immunoglobulin D (IgD) levels (>100 IU/mL) and unexplained recurrent fever [171]. In 1999, two separate international research groups concurrently identified the MVK gene, which is located on the long arm of chromosome 12, as the genetic culprit behind HIDS, establishing a direct association between HIDS and mutations in the mevalonate kinase (MVK) gene [172,173]. This gene is responsible for encoding the ketoglutarate kinase enzyme, which plays a crucial role in the metabolism of cholesterol and homocysteine. This new understanding has significantly contributed to our comprehension of HIDS and its underlying genetic mechanisms.

HIDS is an autosomal recessive disorder in which two alleles of the MVK gene undergo mutation. Together, the four most common variants (V377I, I268T, H20 P/N, and P167L) account for 71.5 % of all mutations found [174]. These mutations in the MVK gene cause disruptions in the ketoacid metabolism pathway, which ultimately lead to a lack of mevalonate kinase, an enzyme essential for the production of nonsterol isoprenoids and cholesterol [172,175]. Therefore, this deficit initiates an inflammatory reaction and abnormal cytokine production. For example, Siroon Bekkering's study showed that in HIDS patients, due to mevalonate accumulation as a result of MVK deficiency, monocytes exhibit “trained immunity”, which leads to increased secretion and significantly higher levels of the inflammatory factors TNF-α, IL-1β, and IL-6; at the same time, IL-1 β, TNF-α and other pro-inflammatory cytokines activate polyclonal B cells, leading to non-specific IgD secretion [176]. Interestingly, HIDS differs from the previous two in that elevated IgD in HIDS patients is a “bystander” phenomenon of chronic inflammation rather than a direct pathogenetic factor. In addition, studies have concluded, on the contrary, that lymphocyte apoptosis is reduced in patients with HIDS and, as a result, the basic function of lymphocytes in controlling the inflammatory response is weakened, leading to an increase in the secretion of pro-inflammatory cytokines [175]. These mutations can lead to two distinct syndromes: HIDS and mevalonic aciduria. Remarkably, HIDS represents a milder phenotype, whereas mevalonic aciduria presents as the more severe manifestation of a single disorder known as mevalonate kinase deficiency. Mutations in the MVK gene result in reduced levels of the MVK protein. The extent of residual MVK activity determines whether a patient exhibits the HIDS phenotype or the more severe mevalonic aciduria phenotype. In HIDS, the residual MVK activity ranges from approximately 1 %–20 %, whereas it is less than 0.5 % in patients with mevalonic aciduria. These findings highlight the critical role of MVK protein activity in determining the clinical presentation and severity of the disease [177].

The pathogenesis of HIDS involves distinctive immunological characteristics, including an aberrantly activated immune system, elevated immunoglobulin D levels, heightened responsiveness to external stimuli, and the release of inflammatory mediators. Collectively, these attributes underlie the recurrent febrile episodes and systemic manifestations observed in individuals with HIDS. A comprehensive understanding of the precise immunological mechanisms driving HIDS remains a subject of ongoing research, necessitating further investigations to illuminate the intricacies of this complex disorder.

### 4.2 Epidemiology

HIDS was initially documented in individuals of Dutch descent by van der Meer et al. [171]. Because HIDS is an autosomal recessive disease, it has no specific preference for either sex. Although the incidence of HIDS does not differ by sex, it varies significantly by region, with the majority of cases occurring in Europe [174,[178], [179], [180]] (Fig. 3(E)).

### 4.3 Clinical manifestations

The median age at initial onset of HIDS is 6 months, and HIDS often starts in early infancy [174]. The most frequent manifestations facilitating initial diagnosis include fever [174], painful lymphadenopathy (>90 %) [170], gastrointestinal manifestations (80–90 %) [168,174], arthralgia (>70 %), arthritis (≈50 %) [181], and skin problems (66.7 %) [175]; while less common but clinically critical features requiring vigilance against misdiagnosis comprise xerostomia, oral/genital ulcers, headache, chills, hepatomegaly, conjunctivitis, amyloidosis (3 %), and macrophage activation syndrome (MAS) [[177], [178], [179], [180]]. (Table 10) (Fig. 3(D)).

**Table 10:** Clinical manifestations in HIDS: Prevalence and characteristic features.

Infections (%)	Infections/Complications (%)
Feverish spells	Fever lasting three to seven days, repeated every four to six weeks
Painful lymphadenopathy (over 90 %)	Primarily localized in the neck, although generalized lymphadenopathy can also occur
Gastrointestinal manifestations (80 %–90 %)	Encompassing abdominal pain, vomiting, and diarrhea
Arthritis (approximately 50 %)	Painful joint swelling
Skin problems (2/3 patients)	Typically presenting as maculopapular rashes, although examples of erythema nodosum, urticaria, and purpura have also been observed
Xerostomia (dry mouth) and the development of oral and genital ulcers	Resembling the symptoms of Behçet's disease
Headache, chills, hepatomegaly, and conjunctivitis	These symptoms are uncommon.
Amyloidosis (3 %)	Specifically AA-type amyloidosis
Macrophage Activation Syndrome (MAS)	Rarely documented, characterized by persistent fever, rash, hepatosplenomegaly, cervical lymphadenopathy, pancytopenia, methemoglobinemia and hypofibrinogenemia
Arthralgia	Arthralgia is another common symptom, affecting over 70 % of patients

∗ Although the interval between febrile episodes varies considerably from patient to patient.

### 4.4 Laboratory diagnosis

The primary laboratory observation involves a persistent elevation of polyclonal IgD [177], mevalonate, pronounced acute phase response and SAA and CRP levels [182]. Compared to the median diagnostic delay in HIGM patients (24 months in Iran) [9], HID and HIE are predominantly diagnosed during childhood, indicating a generally longer diagnostic delay [183,184]. On average, HIDS is diagnosed nearly 10 years after symptom onset, with many patients initially misdiagnosed with conditions such as FMF, adult-onset Still's disease, or juvenile idiopathic arthritis due to the disease's rarity and nonspecific clinical manifestations [184], further contributing to its frequent confusion with other autoinflammatory diseases [185]. A French investigation of 13 MKD patients revealed a range of initially suspected alternative diagnoses, including sJIA, PFAPA, familial Mediterranean fever, chronic infantile neurologic skin and joint syndrome, vasculitis, KD, inflammatory bowel disease, and immunodeficiency disease, prior to the correct diagnosis [186]. Given the rarity of HIDS and the limited, time-consuming, and expensive nature of genetic testing, accurately identifying patients who truly need such diagnostic measures is crucial [177]. In a cohort of 60 patients who experienced recurrent fever and tested negative for the most common autoinflammatory syndromes other than HIDS, no mvk mutations were detected [187]. Only 2 % of patients with recurrent fever, negative FMF test findings, and at least one of the three main characteristics of HIDS (cervical lymphadenopathy, postvaccination reaction, and orofacial stomatitis) had mvk mutations, according to another study [188] (Table 11).

**Table 11:** HIDS laboratory diagnosis: Biomarker targets and diagnostic characteristics.

Targets	Features, characteristics
Elevation of polyclonal IgD	The median IgD concentration typically is approximately 400 U/mL.
Mevalonate	During an attack, trace amounts of mevalonate may be detected in the plasma, albeit the increase is minimal.
Pronounced acute phase response	It is characterized by increased erythrocyte sedimentation rate, granulocytopenia, and elevated serum c-reactive protein (CRP) and SAA concentrations.
SAA and CRP levels	Between episodes, about half of the patients showed signs of inflammation, as evidenced by elevated SAA and CRP levels, although significantly lower than during the actual episode

∗ Elevated serum IgD levels are not universal, especially in younger patients, and IgD values may be within the normal range.

### 4.5 Treatment

There is no consensus on a standardized treatment regimen for HIDS, but clinical practice suggests that interventions such as nonsteroidal anti-inflammatory drugs (NSAIDs) can provide stage-specific control and symptom reduction, in addition to a number of potential therapeutic directions that remain to be identified [[189], [190], [191], [192], [193], [194], [195], [196], [197], [198], [199], [200], [201], [202], [203], [204], [205], [206]]. (Table 12).

**Table 12:** HIDS management: Therapeutic approaches and targeted treatment mechanisms.

Treatment method	Specific treatments
**Existing**	
Taking nonsteroidal anti-inflammatory drugs (NSAIDs)	Blocking the release of inflammatory mediators by taking nonsteroidal anti-inflammatory drugs (NSAIDs), such as ibuprofen and naproxen, can reduce the pain and fever associated with HIDS.
Anti-tumor necrosis factor (TNF) inhibitors	TNF inhibitors downstream pro-inflammatory signaling pathway activation by specifically binding to and neutralizing TNF-α molecules and blocking their binding to cell surface receptors. Used in the treatment of autoimmune diseases such as rheumatoid arthritis and Crohn's disease.
Interleukin-1 Blocking Drugs	IL-1 Blocking Drugs such as anakinra (recombinant IL-1 receptor antagonist) and canakinumab (anti-IL-1β monoclonal antibody) are effective in suppressing systemic inflammatory responses by directly blocking the IL-1 signaling pathway. Several clinical trials have confirmed that these two drugs can significantly reduce the frequency of fever in patients and lower the levels of inflammatory markers such as C-reactive protein (CRP). They are now dual-approved by the FDA and EMA as first-line options for HIDS management.
**Potential**	
Geranylgeraniol (GGOH)	Exogenous geranylgeranylgeraniol (GGOH) re-establishes the membrane localization function of RhoA proteins and restores phosphorylation regulation of Pyrin inflammasomes by PKN1/2 kinases through replenishment of geranylgeranylpropyl phosphate (GGPP) metabolic pool. In vitro experiments demonstrated that this therapy corrected RhoA prenylation defects in fibroblasts from HIDS patients; animal models initially verified its safety and provided theoretical support at the molecular level for clinical translation.
Bryostatin-1 and Arachidonic Acid	The macrolide Bryostatin-1 was found to enhance PKN1/2 kinase activity and promote Pyrin protein phosphorylation, thereby reducing IL-1β secretion in peripheral blood mononuclear cells of patients. While the dietary supplement arachidonic acid possesses a similar mechanism theoretically, its clinical efficacy still needs to be further verified.
Allogeneic hematopoietic stem cell transplantation (HSCT)	HSCT may be the ultimate therapeutic option for patients with severe HIDS who have failed conventional anti-inflammatory therapy. However, the risk of graft-versus-host disease and infectious complications associated with this therapy is significant, and clinical decisions need to be made based on a combination of key factors, such as the patient's functional status and the degree of response to prior therapy, in order to avoid the risk of irreversible damage or graft failure.
Glucocorticoids	Glucocorticoids significantly enhance the bioluminescent activity of the MK-V377I-nLuc reporter system by activating its receptor signaling pathway. The hormone specifically upregulates mevalonate kinase (MVK) expression and activates the sterol regulatory element binding protein 2 (SREBP-2)-regulated metabolic gene network. Based on this, reconfiguring the isoprenoid metabolic pathway homeostasis by targeting the glucocorticoid receptor or directly regulating the SREBP-2 signaling axis may provide a new direction for precision therapy of MKD-HIDS.

∗ Colchicine is also an anti-inflammatory drug, but many cases have shown that colchicine has little effect on the inflammation caused by HIDS and may even lead to exacerbation of the condition.

## 5 Discussion

Maintaining appropriate immunoglobulin concentrations is crucial to effectively defend against exogenous pathogens. However, the immune system can be dysregulated when the concentration of one or more immunoglobulins is elevated, leading to hypergammaglobulinemia (HGG). Given the rarity of HGG and the limited global record of documented cases, our understanding of these disorders remains limited. The intricate etiology and pathogenesis of HGG have yet to be fully elucidated, and understanding the characteristics, diagnostic methods, and treatment modalities of different types of HGG. This study provides a comprehensive understanding and analysis of various aspects pertaining to the pathogenic genes, clinical manifestations, and laboratory diagnostics of two types of HGG: HIGM and HIES. The pathogenic features of these two conditions are intricately linked to abnormal levels of associated immunoglobulins, suggesting the need for timely diagnosis and subsequent treatment based on the clinical features of immunoglobulin abnormalities, laboratory tests, and associated infections in patients presenting with immunoglobulin level irregularities. The inclusion of HIDS in this review, alongside HIGM and HIES syndromes, is grounded in its unique position as a paradigm of systemic immune dysregulation. While HIGM and HIES represent canonical disorders of antibody class-switching defects and Th2/Th17 imbalance, respectively, HIDS provides a critical counterpoint by illustrating how metabolic perturbations in the mevalonate pathway can drive inflammatory cascades that secondarily influence immunoglobulin dynamics. This juxtaposition underscores the multifaceted interplay between innate immune activation, cytokine dysregulation, and humoral immunity—a nexus with broad implications for understanding immune homeostasis (Table 13). Due to the current lack of comprehensive understanding of these conditions, unclear precise etiologies, and limited effective treatment options, the mortality rates for these disorders are exceedingly high, underscoring the critical importance of preventive measures. The pathogenesis of HIGM, HIES, and HIDS is closely associated with specific gene mutations. Herein, we describe the relevant gene mutations for these three conditions, such as the CD40^−^CD40L gene for HIGM, the STAT3 gene for HIES, and the MVK gene for HIDS, providing a genetic testing direction for the prevention and early diagnosis of related conditions. Genetic mutation testing for neonates or individuals exhibiting relevant symptoms can aid in the prevention and early diagnosis of associated HGG disorders.

**Table 13:** Mechanistic and clinical contrasts between HIGM/HIES and HIDS.

Comparison axis	HIGM/HIES	HIDS	Key scientific insight
**Core mechanism**	Adaptive immune failure • HIGM: CD40L mutations disrupt CSR • HIES: STAT3 loss causes Th17/Th2 imbalance	Metabolic-immune crosstalk • MVK mutations impair mevalonate metabolism leading to increased levels of inflammatory factors such as TNF-α, IL-1β, and IL-6.	HIDS exemplifies innate immune dysregulation via metabolic stress contrasting adaptive defects in HIGM/HIES
**Immunoglobulin drivers**	Direct pathogenesis • Elevated IgM (CSR failure) • Elevated IgE (STAT3-dependent cytokine dysregulation)	Indirect biomarker • IgD elevation reflects polyclonal B-cell activation from inflammation	IgD in HIDS is a bystander of inflammation challenging immunoglobulin-centric diagnostic paradigms
**Therapeutic strategy**	Restore adaptive immunity • CD40L agonists/gene therapy • STAT3 modulators	Suppress innate hyperactivation • IL-1 inhibitors • TNF-α antagonists	Therapeutic divergence reflects mechanistic roots: immune reconstitution vs inflammatory pathway blockade
**Diagnostic classification**	Canonical antibody deficiency • Defined by Ig quantification/functional assays	Autoinflammatory disorder • Relies on MVK genotyping/inflammatory markers (CRP/SAA)	HIDS challenges rigid diagnostic boundaries urging integration of metabolic and immune metrics
**Translational implication**	Precision immune reconstitution • Gene therapy/HSCT	Metabolic-immune axis targeting • Mevalonate pathway modulators • Novel anti-inflammatory targets	HIDS provides a model for studying immune-metabolic interplay in rare diseases

Although the incidence of these three conditions may be exceedingly low, their associated mortality rates are alarmingly high, particularly manifesting at an early age, with HIDS demonstrating symptoms within weeks of birth. The impact of these diseases is devastating for countless families, potentially leading to a decrease in population growth, and may even diminish the desire for procreation among many couples. Bolstering research, understanding, and development in this area is imperative to prevent further persecution of affected individuals and to ensure that patients with HIGM, HIDS, and HIES receive more effective and timely treatments. We hope that this article will serve as a conduit for raising awareness and understanding of these conditions, thereby increasing vigilance and preventive measures. Furthermore, we aspire for this work to inspire and guide researchers in their exploration of these diseases.

## 6 Conclusion

A detailed understanding of the causes, genes, symptoms, epidemiology, diagnostics, and treatments of Hyperimmunoglobulin M syndrome (HIGM), hyperimmunoglobulin E syndrome (HIES), and hyperimmunoglobulin D syndrome (HIDS) provides a structured framework for preventing, detecting, diagnosing, and treating these disorders. This review not only guides the management of these conditions but also supplies data for future research into these and other related diseases. The integrated insights emphasize the need for ongoing research in immunology and clinical medicine. In summary, while we understand these three diseases, research remains incomplete, particularly with respect to their pathogenesis, complexity, and treatment limitations. Future research should explore the functions of immunoglobulins, develop accurate disease models, and create new therapies, such as targeted, gene, and cell therapies, to improve outcomes. Interdisciplinary collaboration and public awareness are crucial to advance research and understanding of immunoglobulin-related diseases.

## 7 Limitations

This review has several limitations that warrant acknowledgment. First, the reliance on PubMed as the sole literature source may have introduced selection bias, as studies indexed in other databases (e.g., Embase, CINHAL, or regional repositories) were excluded. While PubMed remains a robust platform for biomedical research, its coverage of non-English or region-specific studies is limited, potentially omitting relevant findings from non-indexed journals or languages other than English. Second, the absence of primary data restricts the analysis to aggregated outcomes reported in existing studies, which may mask underlying heterogeneity in patient populations, diagnostic criteria, or intervention protocols. For instance, variations in study designs (e.g., retrospective vs. prospective cohorts) across the reviewed articles could influence the generalizability of conclusions. To address these limitations, future research could adopt a multi-database search strategy (e.g., combining PubMed with Embase and Scopus) to enhance comprehensiveness. Additionally, incorporating primary data collection through prospective registries or standardized case reporting would strengthen causal inferences and reduce reliance on heterogeneous secondary data.

## CRediT authorship contribution statement

**Wushu Chen:** Methodology, Project administration, Conceptualization, Writing – original draft. **Xingpei Li:** Methodology, Project administration, Writing – review & editing, Writing – original draft, Formal analysis. **Junye Mai:** Writing – original draft, Conceptualization, Formal analysis, Methodology. **Kailang Tang:** Writing – review & editing, Conceptualization, Formal analysis, Methodology. **Yingqiao Wang:** Validation, Conceptualization, Methodology, Formal analysis. **Yan-yan Lu:** Writing – review & editing, Data curation, Conceptualization, Formal analysis. **Jie Liang:** Data curation, Methodology, Conceptualization. **Ni-jiao Li:** Formal analysis, Writing – review & editing, Data curation, Conceptualization. **Xiu-Yu Qin:** Data curation, Formal analysis, Conceptualization, Methodology. **Yu Li:** Resources, Data curation, Formal analysis, Writing – review & editing, Conceptualization. **Lunkai Yao:** Writing – review & editing, Data curation, Formal analysis, Conceptualization, Funding acquisition, Methodology. **Ye Qiu:** Writing – review & editing, Project administration, Data curation, Conceptualization, Resources, Funding acquisition.

## Funding

This study was supported by grants from the 10.13039/501100001809National Natural Science Foundation of China (grant numbers NSFC 82202544), the 10.13039/501100004607Guangxi Natural Science Foundation (No. 2021GXNSFBA220064), and the 10.13039/501100002858China Postdoctoral Science Foundation (No. 2023M730801).

## Declaration of competing interest

All authors disclosed no relevant relationships.

## Data Availability

No data was used for the research described in the article.
